# Collectanea

**Published:** 1830-10

**Authors:** 


### COLLECTANEA.

**Floriferis ut apes in saltibus omnia libant,**

**Omnia nos, itidem, depascimur aurea dicta.**

### PATHOLOGY.

*Case of Poisoning from Nux Vomica.* By GEORGE WATT, Member of the Faculty of Physicians and Surgeons, Glasgow. (*Glasgow Med. Journal.*)

ON the 1st of April last, I was requested to visit Mary Mac——, aged forty, spinster, who was said to have taken poison. I found her lying upon her back on the floor, her arms extended, and removed from the trunk; the inferior extremities likewise in a state of extension, and separated from each other. Her countenance was rather anxious, but she appeared perfectly rational, apparently repenting of the rash act she had committed, and earnestly entreating me to try to save her life. She replied coolly to my interrogations; said she had taken three-pence' worth of nux vomica about an hour before, and that she suffered no pain either in stomach or head. I attempted to give her, by teaspoonsful, two scruples of the sulphate of zinc, dissolved in a little water, but she was unable to swallow, and supposed the difficulty was owing to the awkward posture in which she lay.

On endeavouring to raise her, in order to facilitate the swallowing of the emetic, severe spasmodic action was brought on. The neck and back were thrown into a state of tetanic stiffness, and turned backwards, the arms thrown out from the body, and forcibly extended; the hip and knee joints extended, and so stiff that flexion was utterly impossible. The countenance became at first exceedingly anxious, expressing a state of suffering, then the eyeballs became fixed, and seemed as if about to start out of their sockets, with the jaws locked, and the lips violet coloured, from respiration being obstructed by the spasm of the muscles of the chest, which, with those of the abdomen, were hard and tense; whilst the whole body was agitated with a tremulous movement. Being laid down, the spasmodic action diminished in two or three minutes: she was still unable to swallow, but, anxious for relief, requested to be turned slowly round upon her side, the attempt at which brought on a return of the tetanic spasms; and, on laying her again upon her back, the respiration could not be perceived, and the pulsation of the heart was gone, although, from slight tremulous movements, life did not seem to have left her entirely for nearly a minute.

About half an hour before I was sent for, her mother called in several of her neighbours, to whom she at once told what she had done, without the slightest appearance of remorse, and she did not appear to have been drinking that morning. At that time she made many efforts to vomit, but without effect. These efforts, however, had ceased before I saw her. When put to bed, the convulsive tremors were slight, with intervals of almost complete cessation of spasms. These intervals soon became more and more short, and the spasms so severe that the women about her could not prevent her falling out of bed upon the floor, and it was only then they first thought of sending for medical assistance.

It was nearly sixty hours after death before leave was obtained to open the body. On removing the skullcap and dura mater, the vessels of the pia mater did not appear enlarged or distended: a considerable quantity of serum was effused over the whole superficies of the brain, and likewise upwards of a teaspoonful in each of the lateral ventricles. The substance of the brain, though soft, (I thought more so than usual at the same period after death,) was natural. The lumbar portion of the spinal cord was much softer than the cervical, and the veins on the posterior surface were considerably engorged, for about two inches. The lungs presented less sanguineous engorgement than is usually found in inspections. The heart was flaccid, the ventricles and auricles completely emptied of blood. The stomach contained about a pint of thick dark-coloured fluid, with a fetid smell; the tunics were very easily torn, and in several parts the internal surface presented a marbled appearance. The commencement of the duodenum was so enlarged as to present the appearance of a second stomach; the muciparous glands were enlarged, but there was no softening of the mucous membrane.

Mr. Graham, lecturer on chemistry in this city, examined the contents of the stomach. "The extract (which he obtained) was intensely bitter, and had the smell of liquorice, a property of nux vomica. A solution of the extract in water gave a precipitate with ammonia, but was not reddened by nitric acid. These last properties, however, although the only tests of strychnia which Orfila and Barruel suggest, are by no means characteristic of that principle: that of being reddened by nitric acid, is of an accidental kind. I would rely more upon the taste and odour of the extract."

In this case, death was evidently more immediately caused by the suspension of respiration during the tetanic paroxysm. After death, the rigidity of the body rapidly disappeared. This rigidity has been supposed capable of passing imperceptibly into the stiffness of death, without any relaxation being observable. It is likewise supposed that, in cases of poisoning from nux vomica, the pain and suffering is dreadful. In the above case no pain was complained of during the intervals of the spasmodic paroxysms.

If in similar cases the surgeon is called in sufficiently early, (which will seldom be the case where self-destruction is premeditated,) the prompt administration of an emetic may remove the poisonous matter from the stomach, to the parietes of which, when in powder, it is supposed to adhere very keenly. To wait till all the necessary appendices of a stomach-pump can be procured, would too often cause a loss of time in a case where every second is of the utmost consequence. In one case on record the surgeon went unprovided with an emetic, and found his patient without any bad symptoms: he left her in order to procure one, and, on his return very soon after, found her *in articulo mortis*. The efficiency of the emetic will depend very much on the state of the stomach at the time. If it contains a quantity of still indigested food, there is a greater probability of the whole of the poison being again rejected. If empty, the effects of the poison will be more immediate on the system, and probably, before a surgeon can be called, the spasms have become so severe as to prevent swallowing altogether. In the first condition of the stomach, the evacuation of its contents will be much more easily accomplished by the action of an emetic assisted by tepid drinks, than by the use of a stomach-pump, and with a greater probability of the stomach being completely cleared out. In the second case, it seems to me very doubtful whether the attempt to introduce the tube would not bring on such an attack of spasm as would render its completion impossible. The swallowing of the emetic in the above case, I suspect, was prevented by spasms of the œsophageal muscles, brought on by the attempt; and, if such resistance is made to the passage of a fluid, the introduction of a tube must meet with still more. If, however, the tube of the stomach-pump has reached the stomach, as iodine has been found a sort of antidote to its alkaloid preparation, I conceive that, in some cases, advantage might be found from washing out the stomach several times with tepid water, in which a quantity of tincture of iodine had been agitated. But let it be remembered that the success of iodine seems to depend only on its being administered in two or three minutes after the poison has been taken.

---

*On Polypus of the Uterus, attended by unusual Circumstances.* (Dr. Gooch *on Diseases of Women.*)

In a vast proportion of cases, to speak in numbers, in nine out of ten, to whatever part the stalk grows, frequent hemorrhages from the uterus form the symptom of the disease, which attracts the chief attention of the practitioner, and the case is long mistaken for common menorrhagia; but when the tumor grows from the neck or lip of the uterus, it sometimes occasions nothing but an obstinate and profuse leucorrhœa. The following case shows this, and shows also how liable diseases of this part are to be overlooked for want of examination by touch.

I was sent for, one evening, to see a lady about thirty years old, and was told that she had been troubled for nearly two years with profuse leucorrhœa. As she was unmarried I was going to prescribe for her without examination, when I was told this would not be satisfactory. It then came out that she had seen another physician that morning, whose statement appeared to her and her mother so incredible that they would not believe it until it was corroborated by a second medical witness; but I was not allowed either to meet this physician, or to know what his statement had been. Little as I expected it, I found a small polypus, about the size of a walnut, growing by a slender stalk to the cervix uteri. The ligature was successfully applied, and the patient recovered.

Women who have a polypus of the uterus, especially if it grows from the neck or lip of this organ, sometimes become pregnant. Of this I have known two instances. In one the tumor was discovered in the fifth month of pregnancy, and was removed by the ligature. The pregnancy went on to the ninth month, when the patient was safely delivered. In the other case the tumor was not discovered till the commencement of labour, and occasioned the death of the patient a few hours after delivery.

Mr. Borrett, surgeon at Yarmouth, with whom, when this happened, I was residing as pupil, was called to a lady in labour with her sixth child. On his first examination he found a large fleshy tumor within the vagina. The anterior segment of the os uteri was easily felt, but the posterior was occupied and covered by the attachment of the tumor. After the orifice had dilated and the membranes had burst, the head of the child not descending, Mr. Borrett introduced his hand, brought down the feet, and extracted the child. The placenta was expelled spontaneously. The patient now being delivered, and easy, he left her at seven in the morning. At three in the afternoon he found her in strong pains, as if there was another child; but, as the abdomen was flat, and the contracted uterus could be distinctly felt in the abdomen, he was satisfied that there was not, and gave her an opiate. At eight o'clock at night, he found that the pains continued violent, with a sensation as of a substance coming away, and on examination he discovered a soft round tumor pressing against the outer orifice. What could it be? He would have thought that it was the uterus inverted, but it was the same tumor which he had felt in the morning before the child was born; there was no hemorrhage; the placenta had been expelled spontaneously, and the uterus was distinct in the hypogastric region. He consulted his medical friends in the town, and sent off to Norwich for Mr. Rigby. The pains continued with violent expulsive efforts all night, and the next morning they found her with a languid pulse and a pallid countenance ; a large fleshy livid tumor had been forced out of the vagina, and every pain brought it more and more in sight: she continued to suffer and to sink through the rest of the day ; in the evening Mr. Rigby arrived, bnt she had expired about half an hour before. As soon as he arrived, he examined the tumor, and was convinced that it was the inverted uterus. On opening the body next morning the uterus was found contracted, but its orifice was dragged down as low as the external orifice, by a tumor which grew from it by a thick stalk; it was attached to the posterior part of the orifice, and some way up the neck, was of a livid colour, and weighed three pounds fifteen ounces.

What would have been the result of this case, if a ligature had been applied round the stalk of the tumor, and its body cut off just below, as in the case No. V?

When the polypus grows from the lip of the uterus, and its stalk is very thick, as two or three inches in diameter, the other lip and the orifice of the uterus are quite lost, and cannot be detected by touch. In this case, of which I have seen several instances, instead of feeling a slender stalk encircled by the orifice of the uterus, or the stalk and the orifice by the side of it distinct from one another, we feel at the top of the vagina, where the neck of the uterus usually projects into its cavity, a thick stalk which lower down enlarges into a globular tumor, without any vestige of the neck or orifice of the uterus. I have seen the most experienced practitioners in London puzzled to tell what was the nature of the tumor, and what ought to be done for it.

The following case is a specimen of this puzzling combination of circumstances.

A lady, forty years of age, who had never been pregnant, came to London for advice for frequent hemorrhages from the uterus, which had not been relieved by the usual remedies, and had greatly injured her health. On examination there was found in the vagina a large round tumor, terminating in a very thick stalk, which grew from the situation of the neck of the uterus. This stalk, however, was not encircled by the orifice of the uterus, either completely, as in polypus of the fundus uteri, or partially, as in polypus of the neck. No orifice could be felt. On passing the finger round the uppermost part of the stalk, it formed, with the adjoining part of the vagina, a blind corner all round, as is felt in passing the finger round the neck of the uterus. For some time the nature of the tumor and the propriety of an operation were much doubted, and on this point I was consulted. On carefully examining it, I felt a little depression at the uppermost and anterior part of the stalk. This I conjectured was the orifice of the uterus, small, from the patient never having been pregnant, and dragged out of its natural direction, and obscured by the thickness of the stalk. That a thick stalk growing from the edge of the orifice might so obscure that orifice as to make it difficult to be felt, particularly in a woman who had never been pregnant, seemed intelligible and probable. The tumor was insensible, smooth, and of the consistence of a polypus. Thus it appeared so reasonable to believe that the tumor was a polypus, so certain that if it was not removed the patient would ultimately die of the hemorrhages, and so probable that the operation would be successful, that, notwithstanding the obscurities of the case, I advised it to be performed: the ligature was applied to the stalk, and it was gradually tightened every day. On the fourth day the ligature gave way, and the instrument came off without the tumor: another ligature was now placed in the groove left by the former, and gradually tightened every day. On the fifth day, having made its way completely through the stalk, it came away with the instrument, leaving the tumor loose in the vagina. It was necessary to introduce the whole hand, in order to extract the tumor, which was found to be as large as the end of a new-born child. On examining the vagina a short time afterwards, the neck of the uterus was found in its natural situation, and with its natural orifice. The hemorrhages ceased, menstruation became regular, the patient rapidly recovered, and has been well ever since.

Some years before this case occurred I was consulted, together with two of the most experienced practitioners in London, about a lady who had been long subject to hemorrhages from the uterus, by which her health had been broken, and in whom a large round tumor had been discovered in the vagina. On examining it carefully, the orifice of the uterus could be felt, the anterior lip of which seemed as if it was prolonged into a thick stalk which terminated in a round tumor. As the stalk was not encircled by the orifice of the uterus, it was thought that it could not be a polypus, and we agreed in dissuading from the application of the ligature. This lady died some years afterwards. If I had known what I do now about the thick-necked polypus of the lip of the uterus, and that there is a large proportion of removable polypi, of which the stalk is not encircled by the orifice of the uterus, I would have applied the ligature.

A polypus may have an unusual form, as that of a solid cylinder; it is generally globular or pyriform, with a stalk like an elastic gum bottle; but I have seen two cases in which it was cylindrical, about the size of half the wrist, and the unusual form occasioned great doubts about the nature of the case and the mode of treatment.

A woman for a long time had suffered frequent hemorrhages from the uterus, which had resisted the usual remedies and greatly injured her health. At length, one day, a substance of a singular appearance suddenly protuded from the vagina; it hung out beyond the external orifice nearly half a foot; in form it was like a flattened cylinder, about half as thick as the wrist; it felt somewhat like an intestine, but on careful examination was found to have no cavity; it was very soft; on tracing it upwards it was found to extend through the vagina, and through the orifice of the uterus, beyond which it could not be traced; it was firmly attached within, for on pulling at it, it did not come away; it was of the same diameter through its whole length: a ligature was applied to it just below the orifice of the uterus, and in a few days it came away, the patient immediately losing the hemorrhages by which she had so long been weakened.

A polypus is sometimes so small, it seems incredible that it should occasion the frequent hemorrhages which attend it, yet the hemorrhages cease on the removal of the polypus. I have felt them as small as a filbert without its shell, growing to the neck or lip of the uterus; they were so small that, on being touched, they slipped into the orifice of the uterus, and there remained concealed till the finger was withdrawn and the patient sat up, when they dropped down again into the vagina.

I saw an elderly woman with a polypus of this size: the day was fixed for its removal, but before it arrived, whilst she was using a lotion with a long pewter syringe, it fell away, and from that time the hemorrhages never returned.

Dr. Babington took me into the country one evening with my instrument, to remove a small polypus about the size of a walnut, which he had discovered in a lady the day before. He had described the case to me about a week before, when I advised him to examine the uterus, and said I thought that he would find a polypus. She was about fifty-five years of age, and for a year and a half had been subject to frequent hemorrhages from the uterus without pain. We found her in bed, ready for the operation. The polypus was so small that I took it between my fore and middle fingers and drew it downwards, on which the stalk broke and the little polypus came away. Dr. Babington, on examining immediately afterwards, was surprised to find no polypus, on which I showed it him in my hand. From this time the hemorrhages never returned. These little polypi are generally too small for the ligature, and if they cannot be pulled away with the finger, should be twisted off by a pair of surgeon's dressing forceps.

---

### PRACTICAL MEDICINE.

*Croton Oil in Tetanus.* From Mr. Lawrence's Lectures; quoted by Dr. Short, in his Essay on Croton Oil.

About three years ago I was called to a case of Tetanus, in which there was very great danger, and where the following plan of treatment was completely successful, although the case, at the first view, seemed a very unpromising one.

It was the case of a gentleman, who was about fifty years of age, of a very robust and full habit, accustomed to free living, a man of a very active turn of mind, and who had various and important business on his hands. He was pursuing his avocations in a very active way at the time he met the accident that led to the tetanus, which was in the hottest season of the year. He was riding when, his horse having fallen, he was thrown forward, and his face coming to the ground, he grazed the dorsum of his nose against the gravel of the road, so as to make a slight wound there: that was the only actual wound he received. He thought so little of the accident, that he did not discontinue his ordinary pursuits, nor change his ordinary mode of living, which was, as I said, rather a free one. I do not know that he did more than put a bit of brown paper, with vinegar, on the grazed part. However, although the injury was very slight, yet, at the end of about ten days, when the wound was just getting well, on sitting down to dinner, having asked some friends to dine with him on that day, he felt that he could not use his jaw very freely: he felt some difficulty in masticating and swallowing his food: he was at length induced, more in conformity to the solicitations of his friends than in accordance with his own feelings, to send for a medical gentleman, who took a little blood, and gave him some opening medicine.

Next day he was very much worse, and I consequently saw him. At this time the symptoms of tetanus were very manifest: there was difficulty in opening the mouth, and in masticating; pain behind the sternum, extending through to the spine; his pulse full and strong; his bowels had, however, been pretty effectually relieved by the opening medicine he had taken the day previous. I told him that he should go to bed immediately; for he was then sitting up, as if he had been going about his ordinary avocations. He was very largely bled, and I prescribed very active medicines. The blood was found next day to be buffed and cupped, the opening medicine had acted freely, and he was better. I prescribed a repetition of venesection and a continuation of the aperients, being desirous of producing a still further action on the alimentary canal. He took a pretty large dose of calomel and jalap, which was followed up by a purgative draught composed of senna and salts, but on this occasion no effect was produced by them.

It being a few miles from town, I did not see him in the evening, but the medical gentleman in attendance gave him a large quantity of castor oil, which did not act; he also administered a clyster, but neither did that operate. By the time, then, that

I saw him next day, all these medicines had not acted, and all the symptoms were considerably worse: the neck and back had become affected. I therefore immediately directed the administration of the croton oil. He took a single drop in a teaspoonful of light gruel; and within about an hour a most violent action was produced in the bowels. He discharged such a quantity of matter, of various kinds, from them, as altogether astonished him and all those about him. They seemed to be quite at a loss to know how to describe the quantity. It seemed he had filled and overfilled the closestool pan, and that so abundant a discharge had taken place from the bowels as was never before witnessed. This was followed by considerable relief of all the symptoms; but still the complaint went on; and, in fact, it proceeded to the full developement of tetanus over the whole body.

The treatment of the case consisted from this time in the regular administration of the croton oil, so as to secure a continued action of the bowels. In the first instance a drop of the oil did this; but after a time or two more, it required a drop and a half, and then the evacuations were very copious indeed. It was observed by the gentleman in attendance, that, if ever he had to prescribe any thing again in the shape of antispasmodics, he would exhibit the croton oil, because it had, in this instance, a much greater effect than any thing he had ever seen; the muscles, which before were very rigid, being soon very much relaxed: indeed, the patient acknowledged, although greatly depressed and weakened by the effect, the power it had in relaxing the rigidity.

I need not detail the whole of the particulars of this case: it is enough for me to say that the tetanic affection proceeded until all the parts of the muscular system were involved; and they were as severely involved as in any case I ever saw. This gentleman frequently remained with all the muscles of the trunk and limbs in the most violent state of rigidity; he then had convulsions of the most serious kind, so that, although he was a man of strong mind and great resolution, he could not at times help crying out most loudly.

It became necessary afterwards to exhibit opium; and the learned lecturer concludes by stating, that "by the continuance of the two remedies, the regular exhibition of the croton oil and the opium, the complaint gradually subsided, and this gentleman completely recovered."

---

*Web of the Black Spider in Delirium Tremens.* (WRIGHT *on Delirium Tremens; American Journal of Med. Sciences.*)

The web of the black spider has received commendation from many respectable sources as a sedative agent, capable of calming, with peculiar ease and certainty, morbid excitability of the cerebral and nervous systems. On the credit of those qualities it has been employed in the various forms of temulence, and not without a share of reputed success, sufficient to entitle it to consideration in that state of constitutional irritation. In the summer of 1827, we tried this article in many cases, and in full doses. To test its qualities, it was given, where the state of the patient admitted, uncombined with opiates. When thus used, its effects were generally partial or doubtful, and its powers inadequate to the production of tranquillity or sleep. In one case only, have I found this substance to exert great or decided sedative attributes: this was the case of an intelligent young man, (in private practice,) who, after consuming, by his own report, three quarts of brandy in thirty-six hours, fell into a state of temulent excitation so excessive that he was incapable of keeping a recumbent or even a sitting posture for more than a moment, but paced his chamber with a ceaseless step for two days and nights. He was not delirious: on the contrary, his conversation was rational, though hurried and vehement. But he was so far under the influence of spectral hallucination, that if he closed his eyes for a moment, day or night, he was instantly visited by a host of phantoms of frightful aspect;* hence chiefly his aversion to lie down, or make any voluntary effort to sleep. This patient took opium, opium with camphor, and black drop, at short intervals, and in full doses, until the quantum of opiate approached the utmost limit of probable safe administration, without even partial relief of constitutional irritation, or any apparent proneness to sleep. The temulent excitement kept unabated for twenty-four hours, the second night passed in constant vigilance, locomotion, and mental excitement, and it seemed probable that excitation so intense, protracted, and unremitting, must soon lapse into delirium or convulsions. At this time, the morning of the third day, (the second of my attendance,) he began the use of the fresh web in pills of five grains every hour. Its effect was prompt and unequivocal. He calmed, even sensibly to himself, with every dose, and watched with desire for the time of repeating the pills. The first effect of the web was to abate his restless movements about the room; he became disposed to sit down, and kept his chair, with short intervals of walking, for some hours. In the evening he consented to go to bed, got up once or twice, but returned to bed without difficulty, took an opiate, at eight, the first for eighteen hours, and slept continuously for eight hours. The cure was completed without difficulty, by repeating the web less frequently next day, quiet, suitable nourishment, and another opiate at night. The patient spoke emphatically, both the first and second day, of the soothing influence produced by the pills. He was not at the time informed of their composition.

---

### SURGERY.

*Case of Division of the right Carotid Artery, successfully treated.* By C. B. Garrett, Esq. (*Midland Med. and Surg. Reporter.*)

A poor man, named James Hancock, æt. twenty-four, resident in this neighbourhood, having been engaged and detected in an intrigue of gallantry, laboured under depression of spirits, and thrice attempted suicide by hanging, in consequence of which, fears were entertained by his family that he would again resort to some mode of self-destruction, and probably a more effectual one. On the evening of the 18th of May last, the man borrowed a razor from a neighbour, for the purpose, he alleged, of shaving himself. His parents having received information of this, watched him the more closely, and observed him going into an adjacent apartment, where he put his intentions into execution; but his courage failing him, he immediately ran into the room where the family were, pressing with all his might on the bleeding vessel.

This occurred about eight o'clock P.M., and happening to be at a house within a few yards, I was with him instantly, and found he had made two incisions in a transverse direction, the one about half an inch above the other; the uppermost and first incision extended from the left side of the throat, over the pomum adami, for about two inches; and the other, about three inches in length, cutting deeply into

* Among the delusions practised by imagination on reason, the impulse to self-destruction was predominant; a catastrophe said to have been unhappily realized in less than a year after, while confined in one of the public institutions on account of derangement from drink.

the trachea, and terminating immediately after dividing the right carotid artery and jugular vein; the artery was bleeding profusely, and the stream of blood was of about the circumference of a swan shot. I lost no time in securing the artery by means of a ligature, although it had receded considerably under the integuments. About a minute and a half only had elapsed betwixt the perpetration of the desperate act and the securing of the artery. Nevertheless, the hemorrhage was excessive, and in this short space of time an amazing quantity of blood was lost. The man was now apparently dying; his respiration laborious and protracted; eyes glazed; pulse nearly imperceptible, and a cold diaphoresis exuded all over the surface of the body. I immediately exhibited diffusible stimuli; had his feet put in warm water, and used all means possible to produce re-animation. When vitality was again manifest, I with great difficulty applied sutures to the incisions, owing to the hacked and ragged state of the parts. Having, at length, succeeded, and applied adhesive strips, I discovered venous blood oozing from the right jugular vein, (I imagined,) but it soon ceased. I ordered him weak brandy and water at intervals, and, as soon as possible, removed him to bed, taking care to secure his head from falling back, and so tearing the wounds asunder. I visited him at half-past ten again, and found him rather feverish, at the same time very weak. Discontinued the brandy and water, and, as his bowels had not been moved for two days, administered Olei Ricini ℥vi. and gave a mixture of tincture of henbane and camphor julep every three hours.

I saw my patient at ten o'clock on the following morning, when he complained of pain in the head and drowsiness. The bowels had been freely moved; tongue enveloped in a white coat; pulse weak and quick; considerable pain in the larynx and summit of the trachea; the wounded parts highly inflamed; deglutition extremely difficult; feet and legs cold as death, to which were applied heated bricks, and I ordered an antiphlogistic lotion to the throat externally, composed of tincture of opium, acetate of lead, and water.

I now began to fear that his constitution would not recover from the shock it had sustained, and, as he was gradually becoming more debilitated, I exhibited, every hour and a half, ether, laudanum, and camphor julep; and occasionally sago, with a little sherry in it. He now began to recover strength, and at four P.M. I found him much better, and the pain completely removed from his head. A glow of heat diffused throughout the system, and pulse considerably elevated and more full. Discontinued the draught, and, from that time, gave him saline medicines principally, with calomel and opium at bedtime occasionally.

On the 20th, I removed the dressings, and found the parts in a most healthy condition, and generating a "laudable pus," the inflammatory symptoms having considerably abated. The proper dressings, and the application of the above lotion occasionally, together with a proper attention to regimen in diet, exercise, &c. produced a salutary effect, and the man is now perfectly recovered, owing his life entirely to the immediate assistance rendered, and I have no doubt but that, in half a minute longer, he would have died from loss of blood. He is, however, now quite well, retaining the cicatrix as the only visible demonstration of his imprudence, and, as it were, showing a conspicuous beacon in warning any who may have been inclined to follow his course, to avoid the shoals of dishonour and profligacy, which are the too frequent instigators of the act.

*Remarks upon the application of Ligatures to the Penis, after the method of* M. Græfe. By M. Michaelis, of Berlin. (*Journ. der Chir. und Augenheilkunde.*)

Towards the close of the 17th century, Ruysch and other surgeons adopted the practice of applying ligatures to the penis, but latterly this operation has been neglected. In 1815, M. Græfe revived it. M. Michaelis records four cases which appear to be in favor of the practice. The subject of the first operation was a man twenty-nine years old, who had contracted, from impure connexion, a venereal ulcer upon the glans penis. The ulcer yielded to a course of mercury, but returned in a few months, and then resisted every plan of treatment. It rapidly increased in extent, and sometimes bled very profusely. In about a year after the patient had been first diseased, it was deemed necessary to remove the penis: he was removed to Berlin to be operated upon by Græfe. A catheter was introduced into the urethra, and then, with a strong ligature, the penis was strangulated immediately behind the glans. The patient bore the operation with great sang froid, and without appearing to suffer the least pain. No fever followed. In the evening of the same day the part beyond the ligature was deprived of vitality, and the ligature was tightened without any inconvenience to the patient. On the second day the dead part was detached and the catheter was withdrawn. The cure proceeded so rapidly that in one month the patient quitted Berlin.

Case II. Half the penis was cancerous. The operation was performed as in the last case: but little pain was complained of. In twenty-four hours the sphacelated part was removed, and the cure proceeded favorably, being interrupted only for a short time by an attack of intermittent fever, which occurred the third day after the operation.

Case III. After an old venereal disease, cancer of the penis occurred. The patient felt perfectly well after the operation. No fever followed, and the wound rapidly healed. Ten days after the operation the man quitted Berlin.

Case IV. A man, sixty years of age, was affected with cancer of nearly the whole of the penis. The disease had arisen about a year after several ulcerations which had appeared upon the glans without any known cause, and which, by uniting, had formed one vast ulcer, which extended to near the pubis. In applying the ligature it was necessary to draw the penis firmly forwards, in order that no diseased part might remain. Fifteen days after the operation the patient had perfectly recovered.

---

*Remarkable case of the re-union of a divided part.* In the Quebec hospital Reports we find the following case. A man, in chopping wood, cut off the first phalanx of the middle finger. For two hours after the accident he remained occupied at home. Although the divided portion of his finger then appeared to be deprived of vitality, it was determined to follow the plan of Balfour of Edinburgh, and to attempt to re-unite the parts. The tip of the finger was fixed to the stump by adhesive plaster, and in three days union had taken place in two or three parts, and the extremity of the finger which had been divided had as much sensation as any other part of the body. The dressing was continued, and in three more days the re-union was complete.—*Baltimore Adviser.*

### MEDICAL JURISPRUDENCE.

*Poisonous Gases. Case of Death from inhaling Nitrous Ether Vapour. (Midland Med. and Surg. Reporter.)*

On the 31st of March, an inquest, of an interesting nature, was held on the body of Elizabeth Stephens, at the house of Mr. Thomas, druggist, Hay, Breconshire, before the coroner, C. Ekins, esq. The deceased lived in the service of Mr. Thomas, and went to bed in perfect health the night previously; but did not rise at her usual hour in the morning, in consequence of which, one of the family went to call her, but found the door fastened, which being broken open, she was found dead, lying on the right side, with the arms folded across the breast as in profound sleep, and the features not at all disturbed.

Medical assistance was immediately procured, and as the material facts of the case were given by the medical attendants, before the jury, we shall give the result of their examination, and the verdict, and make such remarks as the circumstances seem to call for.

Richard Procter, surgeon, being duly sworn, stated that he, jointly with Mr. Hathway and Mr. Henry Procter, opened the body of the deceased, and found the coat of the stomach of the deceased a little inflamed, with a small quantity of fluid in it, not exceeding one ounce; there did not appear to be any gritty substance in the stomach. The intestines leading from the stomach appeared turgid. On further examination, the uterus was found enlarged, and its outer coat highly vascular. On its being opened, it was found to contain a male fœtus, indicating that she had been pregnant about three months. Witness further stated that he saw the deceased soon after the body was discovered, and remarked that a large jar, containing upwards of three gallons of spirits of nitrous ether, was broken, and the contents spilt about the room; and the room being small, and the atmosphere being highly impregnated by the said spirit, witness was of opinion that it was sufficient to have caused the death of the deceased.

Mr. Henry Procter stated that he was present at the examination of the deceased, and concurred with the former witness in his opinion as to the cause of death.

Mr. Nicholas Hathway, surgeon, stated that he was present at the examination of the deceased, and found the stomach and uterus in the state described by the first witness. He was further of opinion, that the impure atmosphere of the room, caused by the evaporation of so large a quantity of spirits, may have produced death, as the lungs were found in such a high state of congestion as to prevent the passage of air through their cells. The witness could not account for the inflamed state of the stomach.

The verdict of the jury was, that the deceased died in consequence of the effluvia arising from the nitric ether, as described by the evidence.

*Observations.* It is much to be regretted that in this very rare, and, we believe we may say, unparalleled occurrrence, the examination of the body was confined to ascertaining the state of the lungs and stomach. The condition of the brain, the heart, and of the whole alimentary canal; the state of the blood, and also the appearances of the surface of the body, should undoubtedly have been mentioned, not only with a view of answering the great ends of the inquiry for which the jury was summoned, but also to throw light upon an important toxicological investigation.

There can, however, be no doubt that the state of the body, in this instance, so far as the examination of the lungs was carried, corresponded with the morbid appearances left in the body after poisoning by carbonic acid gas. Dr. Schenck, medical inspector of Siegen, in reporting two cases of death caused by the vapours of burning wood, notices paleness of the countenance as a singular accompaniment of cerebral congestion; and calls the attention of medical jurists to the extreme calmness of the features as a general character of this variety of poisoning. The lungs, too, in all these cases, are distended with black fluid blood; but in addition to this, according to Portal, the vessels of the brain are gorged, and the ventricles contain serum.*

In Wildberg's collection of cases, there is a report on two people who were suffocated in bed, in consequence of the servant having neglected to open the flue trap when she kindled the stove in the bedchamber; and in each of them Wildberg found all the appearances described by Portal. Mertzdorff has related a case of death from the same cause, in which, together with the preceding appearances, an effusion of blood was found between the arachnoid and pia mater, over the whole surface of both hemispheres.†

We cannot, for a moment, hesitate in believing that the spirit of nitrous ether is capable of producing, when inhaled largely, fatal consequences; as its vapour is known to exert all the influence of a powerful narcotic poison upon the system, and we have recently heard of an instance where sulphuric ether, (whose action is said to be less powerful,) being inhaled by a stout man, produced nearly fatal consequences. In this instance the young man, by breathing for some time the vapour of sulphuric ether, fell into an insensible state, and remained almost apoplectic for some hours, and doubtless would have died had he not been removed from the situation in which he was inhaling the poisonous gas. As, however, the co-existence of inflamed stomach and pregnancy, in the case under consideration, was a circumstance in itself likely to raise suspicions as to the manner in which the deceased came by her death, it became the more necessary for the medical witnesses to show, first, that in every particular disclosed by a careful examination of every part of the body, the truth of their evidence was borne out by the investigations of previous examples where poisonous gases had occasioned death; and, secondly, that no morbid traces were discoverable which indicated that any narcotic, or other poison, had been swallowed.

We may here hazard an opinion we have long held, that the inquest system of this country does not work well. Had such a case as the foregoing occurred amongst our continental neighbours, a full and minute report would have been made of all the particulars connected with it, and nothing would have been left to conjecture, which a patient chemical and anatomical investigation could have disclosed. To the neglect in this country of this kind of medico-legal investigation, may be attributed the meager reference to works in our own language on these important topics, whilst our neighbours, both in France and Germany, have succeeded in collecting together a large store of materials, the fruits of long-continued labour and indefatigable research. At this time, when our legal institutions are undergoing a strict scrutiny, which in some instances has led to wholesome reform, it is well worthy the consideration of our enlightened statesmen and lawyers, whether some material improvement might not be effected in the mode of conducting inquiries before the coroner.

* See Dr. Christison on Poisons, p. 602.

† Consult Dr. Christison on Poisons, p. 603.

